# Analysis of the spatial distribution of occupational burnout among mental health professionals at the city, county, and township levels in Guangxi and related influencing factors

**DOI:** 10.3389/fpubh.2026.1914734

**Published:** 2026-08-04

**Authors:** Wenhua Huang, Fanpei Ye, Tao Lin, Zijian Tang, Jianlan Wei, Jiayu Wu, Lidi Lei, Pinghua Zhu, Peng Cui

**Affiliations:** 1 School of Information and Management, Guangxi Medical University, Nanning, Guangxi, China; 2 The Guangxi Zhuang Autonomous Region Brain Hospital, Liuzhou, Guangxi, China; 3 School of Humanities and Social Science, Guangxi Medical University, Nanning, Guangxi, China; 4 School of Marxism, Guangxi Medical University, Nanning, Guangxi, China

**Keywords:** Guangxi, mental health professionals, occupational burnout, spatial distribution, influencing factors

## Abstract

**Background:**

This study aims to examine occupational burnout among mental health workers at the municipal, county, and township levels in Guangxi in 2024, as well as its influencing factors, from the perspective of ensuring workforce stability, thereby providing a theoretical basis for the development of targeted intervention measures.

**Methods:**

This study employed cluster sampling to conduct a cross-sectional survey of 1,520 mental health workers from different regions of Guangxi, China. The Chinese version of the Maslach Burnout Inventory-General Scale (MBI-GS) was used to assess burnout levels. To ensure the stability of the mental health workforce, we conducted univariate and binary logistic regression analyses using SPSS 27.0 (IBM Corp., Armonk, New York, USA) software across three dimensions: demographic information, institutional level, and work recommendations. Additionally, we utilized ArcMap 10.2 (Esri (Environmental Systems Research Institute, Inc.), Redlands, California, USA.) software to visualize occupational burnout levels across cities in Guangxi using hierarchical maps, thereby identifying the key factors influencing occupational burnout among mental health workers in the region.

**Results:**

The prevalence of occupational burnout among mental health workers in Guangxi was 65.46%. Workers in county-level (OR = 2.977, 95% CI: 1.480–5.988) and township-level (OR = 2.312, 95% CI: 1.210–4.416) mental health institutions were more likely to experience occupational burnout. In contrast, mental health workers in municipal institutions exhibited a lower risk of occupational burnout. Based on the recommendations made by mental health professionals, those who suggested “reducing the workload” (OR = 3.449, 95% CI: 2.142–5.554), increasing staff (OR = 2.714, 95% CI: 1.527–4.822), and lowering the difficulty of tasks (OR = 2.745, 95% CI: 1.406–5.359) showed a significantly increased risk of occupational burnout. Among the 14 cities in Guangxi, mental health workers in Laibin, Yulin, Hezhou, and Nanning exhibited higher burnout level than those in other cities.

**Conclusion:**

The prevalence of occupational burnout among mental health workers in Guangxi was relatively high. To ensure the stability of this workforce and reduce staff turnover, the competent authorities overseeing mental health services should prioritize monitoring burnout levels among workers at the county and township levels, and in key urban areas. Additionally, measures such as reducing workloads, increasing staffing, and simplifying tasks can help alleviate and symptoms of occupational burnout.

## 1 Background

The World Health Organization (WHO) defines burnout as a syndrome conceptualized as resulting from chronic workplace stress that has not been successfully managed (1). Burnout refers to a reaction caused by excessive work stress, characterized primarily by emotional exhaustion and a lack of emotional resources—emotional exhaustion; negative and detached reactions toward others, accompanied by a loss of idealism—depersonalization; and a decline in work capacity and performance—reduced personal accomplishment (2). Existing research indicates that healthcare workers are a high-risk group for occupational burnout (3, 4). Occupational burnout can have far-reaching consequences for healthcare providers, such as emotional disorders, family conflicts, reduced self-esteem, and premature departure from the workforce (5). For patients, healthcare workers' occupational burnout may lead to lower quality of care, reduced safety, and lower patient satisfaction (6, 7). Consequently, the issue of burnout among healthcare workers has garnered widespread attention during the COVID-19 crisis (8, 9). Furthermore, while burnout among healthcare workers in key departments and positions, such as emergency departments (10), pediatrics (11), and radiology (12), has received significant attention, the issue of burnout among mental health professionals appears to have been consistently overlooked.

In December 2004, the Chinese government launched a large-scale “Central Government Subsidy Program for the Management and Treatment of Severe Mental Illness at the Local Level,” commonly referred to as the ““686 Project.” “The primary objective of the project is to explore an integrated hospital-community service model for patients with mental illness (13), prioritizing equal access to basic community mental health services, family education and support, and continuous medication for individuals with severe mental illness, particularly for those living in poverty (14). Service providers under the “686 Project” (hereinafter referred to as mental health workers) include various medical professionals and administrators at the national, provincial, municipal, county, township, and village levels of the hierarchy. Among them, municipal-level mental health workers are responsible for the overall planning, technical guidance, staff training, and information system management of mental health work within their jurisdiction. County-level mental health workers implement technical directives from higher authorities and guide grassroots units in conducting patient screening, registration, and follow-up. As frontline implementers of community management, township (subdistrict) mental health workers primarily conduct investigations into leads on suspected patients, implement follow-up interventions and risk assessments based on disease severity, handle emergency responses, and provide guidance on adhering to medication. They are a key force in community prevention and control, as well as in the comprehensive management of severe mental disorders (15).

This mental health prevention team has continued to grow, expanding from 50,901 participants across 113 cities (prefectures) in 2010 (16) to a massive force that covers all of mainland China and serves over 6 million patients (17). Although they are healthcare professionals, compared to those in other specialties, they not only face generally lower compensation but also suffer from discrimination (18) and even the risk of violence from patients with severe mental illnesses. Due to the inherent and external pressures of the profession, the constant need to manage others' psychological distress and medical conditions, occupational burnout, self-stigmatization, social indifference, and easy access to psychiatric medications, mental health workers have become a particularly vulnerable group (19). In contemporary China, where social stability is highly emphasized, addressing their burnout is of critical importance for ensuring the stability of this workforce and preventing incidents of injury or death caused by patients with mental illnesses experiencing relapses.

Although a small number of scholars in China have studied occupational burnout among mental health workers, their research has typically focused on staff at grassroots-level institutions and involved relatively small sample sizes. The novelty of this study lies in its large sample size, which encompasses mental health workers at the municipal, county, and township levels, making it more representative of population. Using a quantitative survey method, this study investigated the occupational burnout status of mental health workers across the Guangxi Zhuang Autonomous Region in December 2024, aiming to analyze factors that may influence their occupational burnout. It is hoped that the findings will provide a reference for improving the working conditions of the mental health workforce, thereby effectively alleviating occupational burnout among grassroots mental health workers as they face increasingly heavy workloads in the future, reducing staff turnover, and enabling them to carry out their work in a sustainable and healthy manner.

## 2 Methods

### 2.1 Participants

This was a cross-sectional survey. Cluster sampling was used in study. The study population comprised all active mental health workers responsible for the service management of patients with severe mental disorders in communities at the city, county, and township levels in the Guangxi Zhuang Autonomous Region of China. Among the respondents, “full-time” refers to those whose primary job is mental health prevention and treatment, while “part-time” referred to those whose primary focus is mental health prevention and treatment but who also managed other chronic diseases. We conducted a large-scale survey through provincial mental health management departments, distributing electronic questionnaires to all mental health workers. The survey was launched and completed in December 2024. According to a report published by the Guangxi Zhuang Autonomous Region Health Commission, there were 1,625 township health centers and community health service institutions in Guangxi during the same period (20). We received 1,569 the completed questionnaires, indicating that approximately 96.55% of mental health workers participated in the survey. Of the returned questionnaires, 1,520 were valid, yielding a response rate of 96.88. Forty-nine invalid questionnaires were excluded due to missing key items, logical inconsistencies in the information, or omissions of core indicators. All participants in the survey signed informed consent forms.

### 2.2 Questionnaire

The Maslach Burnout Inventory General Scale (MBI-GS), developed by Maslach et al. comprises three dimensions: emotional exhaustion (EE), depersonalization (DP), and low personal accomplishment (PA). The original version consists of 16 items (21). This study utilized the Chinese version revised by ChaoPing Li et al., which contains 15 items (22). Emotional exhaustion (EE) and depersonalization (DP) were scored positively, whereas personal accomplishment (PA) was scored negatively. All responses were rated on a 7-point Likert scale, ranging from 0 (“Never”“) to 6 (“Every day”) (23). The mean score for each dimension was calculated by averaging the scores of the items within that dimension. The total MBI-GS score was calculated using the following formula: total score = (EE × 0.4) + (DP × 0.3) + (PA × 0.3). Based on the total score, the severity of occupational burnout was classified as follows: no occupational burnout group (0–1.49 points), mild occupational burnout group (1.50–2.99 points), moderate occupational burnout group (3.0–4.99 points), and severe occupational burnout group (≥5 points) (22, 24). In this study, the scale demonstrated excellent internal consistency and construct validity, with a Cronbach's alpha coefficient of 0.869, a Kaiser–Meyer–Olkin (KMO) coefficient of 0.933, and a statistically significant result on the Bartlett's test (*p* < 0.001).

### 2.3 Data analysis

Statistical analysis was performed using SPSS 27.0 software, and gradient maps were created using ArcMap 10.2 software and the natural breakpoint method. Burnout scores are expressed as the mean ± standard deviation ($\bar{X}$ ± *S*), while categorical data are presented as counts and percentages. The analysis of influencing factors was conducted with demographic characteristics, part-time work status, educational background, professional title, specialty category, years of service, years of experience in mental health prevention work, institutional level, and work recommendations as independent variables, and occupational burnout as the dependent variable. In the univariate analysis, an independent-samples *t*-test was used for two-group comparisons, and one-way ANOVA was applied for multi-group comparisons. Prior to these parametric tests, normality and homogeneity of variance were assessed for total burnout scores. Although the Shapiro–Wilk test revealed mild deviation from normality, the large sample size of this study (*n* = 1,520) ensured that sample means approximated a normal distribution per the Central Limit Theorem (25). Meanwhile, Levene's test confirmed satisfactory homogeneity of variances across all groups (*P* > 0.05); accordingly, parametric tests were retained. For *post hoc* multiple comparisons, the LSD method was adopted under homogeneous variance, whereas Tamhane's T2 was used when variances were heterogeneous. To mitigate Type I error inflation, the Bonferroni correction was applied to adjust the significance threshold for *post hoc* analyses. In the multivariate analysis, general demographic data and “work recommendations” were used as independent variables, with occupational burnout as the dependent variable (no burnout = 0, burnout = 1). A dummy variable was created for “work recommendations” with “no recommendations” as the reference category, and a binary logistic regression model was used to analyze the factors influencing occupational burnout. To prevent the premature exclusion of factors that may be practically significant but only marginally significant in univariate tests, and to minimize the omission of key variables, we included variables with *P* < 0.10 from the univariate analysis into the model to identify independent predictors of occupational burnout. To test the model's stability, we used the variance inflation factor (VIF) to assess multicollinearity among all independent variables. The results show that the VIF values for all variables ranged from 1.03 to 4.27, all well below the critical threshold of 10, indicating that there is no significant multicollinearity among the predictors. In addition, to verify whether converting the continuous total burnout score into a binary variable affects the robustness of our conclusions, we conducted a multiple linear regression analysis as a sensitivity test, using the original continuous total burnout score as the dependent variable and including the exact same independent variables as in the logistic model. The results showed that the core influencing factors and their directions of influence identified by the two models were highly consistent, demonstrating that the binary coding did not alter the study's main conclusions. The significance level was set at *P* < 0.05.

### 2.4 Ethical considerations

This study was approved by the Ethics Committee of the Guangxi Zhuang Autonomous Region Brain Hospital (No.2024-011). All participants provided informed consent and voluntarily participated in the study.

## 3 Results

### 3.1 Demographic characteristics of the study participants

Among the 1,520 participants in this survey, 522 were male (34.34%) and 998 were female (65.66%), with a male-to-female ratio of 0.52:1; a total of 501 (32.96%) held a bachelor's degree or higher, 1,261 respondents (82.96%) were part-time workers, and 997 respondents (65.59%) had been engaged in mental health prevention work for ≤ 5 years. Further demographic data on the study participants, are presented in Table 1.

**Table 1:** Demographic characteristics of the study participants.

Variable	Category	Number (*N* = 1,520)	Percentage (%)
Gender	Male	522	34.34
	Woman	998	65.66
Age (years)	25 ≤	182	11.97
	26–30	249	16.38
	31–35	228	15.00
	36–40	300	19.74
	41–45	247	16.25
	46–50	137	9.01
	>50	177	11.64
Full-time and part-time status	Full-time	259	17.04
	Part-time	1,261	82.96
Education	Bachelor's degree or higher	501	32.96
	Associate degree or lower	1,019	67.04
Professional specialty	Doctor	654	43.03
	Nurse	500	32.89
	Technician	91	5.99
	Pharmacist	136	8.95
	Other majors	139	9.14
Professional title	Beginner	1,177	77.43
	Intermediate	245	16.12
	Advanced	98	6.45
Years of work experience after graduation	≤ 5 years	317	20.86
	6–10 years	331	21.78
	11–15 years	296	19.47
	16–20 years	205	13.49
	21–30 years	241	15.86
	>30 years	130	8.55
Years of experience in mental health prevention and treatment	≤ 5 years	997	65.59
	6–10 years	367	24.14
	11–15 years	111	7.30
	≥16 years	45	2.96
Level of specialized prevention agency	Municipal	48	3.16
	County-level	146	9.61
	Township-level	1,326	87.24
Work recommendations	No suggestions	885	58.22
	Strengthen professional training	138	9.08
	Hire additional staff	82	5.39
	Reduce the workload	137	9.01
	Improve benefits	65	4.28
	Strengthen interdepartmental coordination	86	5.66
	Increase funding	70	4.61
	Reduce the difficulty of the task	57	3.75

“Other Majors” includes civil service, social work, health management, billing clerks, and other related fields.

### 3.2 Chart of the overall mean scores and grades of occupational burnout among mental health workers in various cities

**Figure 1:**
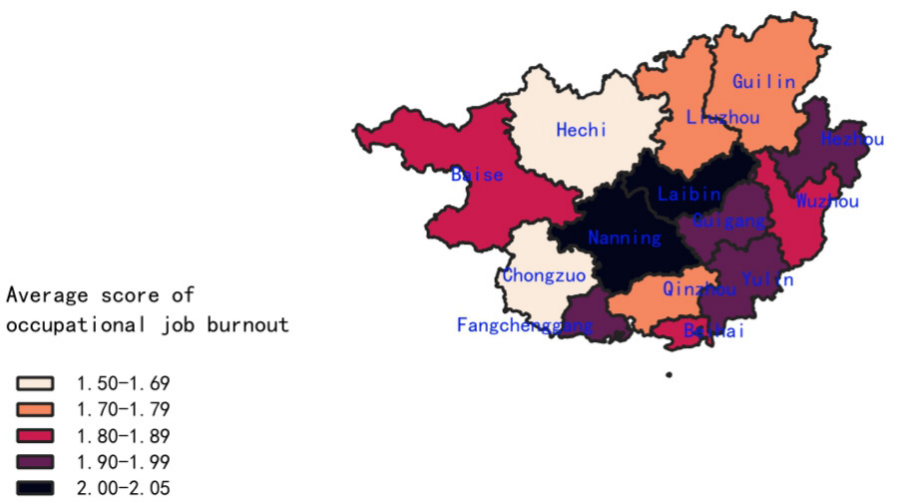
Chart showing the overall average scores for occupational burnout among mental health workers in cities across Guangxi.

This study covered all 14 prefecture-level cities in Guangxi. Among them, Yulin City had the largest number of mental health professionals at 284 (18.68%), followed by Guilin City with 178 (11.71%), and Qinzhou City had the fewest at 23 (1.51%). Table 2 and Figure 1 show for the overall mean scores and grade distribution of occupational burnout among mental health professionals in various cities across Guangxi.

**Table 2:** Burnout scores among mental health professionals in various cities in Guangxi.

Region	Number of examples	Overall average ($\bar{X}$ ±*S*)
Nanning City	153	1.98 ± 1.16
Liuzhou City	127	1.70 ± 0.93
Guilin City	178	1.68 ± 0.91
Wuzhou City	76	1.81 ± 1.06
Beihai City	37	1.81 ± 0.99
Fangchenggang City	39	1.88 ± 0.88
Qinzhou City	23	1.70 ± 1.07
Guigang City	72	1.88 ± 1.15
Yulin City	284	1.95 ± 1.00
Baise City	153	1.77 ± 0.97
Hezhou City	61	1.96 ± 0.84
Hechi City	149	1.46 ± 0.78
Laibin City	74	2.01 ± 1.09
Chongzuo City	94	1.66 ± 0.88

### 3.3 Screening for job burnout and univariate analysis of associated factors

In this study, occupational burnout detection rate was 65.46%, with 56.05% of participants exhibiting mild occupational burnout and 9.41% experiencing moderate to severe occupational burnout. The average occupational burnout score among participants was 1.83 ± 0.99, meeting the criteria for mild occupational burnout (1.50–2.99 points). The prevalence of occupational burnout showed statistically significant differences (*P* < 0.05) across eight factors: age, full-time/part-time status, educational attainment, professional category, years of work experience since graduation, years of experience in mental health prevention work, institutional level, and work advices. An independent samples *t*-test was conducted on the two subgroups with significant differences, revealing that among full-time and part-time staff, part-time staff exhibited significantly higher burnout levels than full-time staff; Bonferroni *post-hoc* multiple comparisons were performed for variables with three or more subgroups: regarding age, the group aged >50 years exhibited significantly lower burnout levels than all other age groups, while the 26–30 and 36–40 age groups showed significantly higher burnout levels than the ≤ 25 age group; regarding professional category, nurses and “other professionals” exhibited significantly higher burnout levels than physicians; In terms of years of experience in mental health prevention and treatment, the burnout level in the 11–15-year group was significantly lower than that in the ≤ 5-year group; regarding “work recommendations,” reproductive health prevention staff who suggested “increasing staff,” “reducing workload,” and “lowering work difficulty” had significantly higher burnout levels than all other feedback groups, while the “no suggestions” group had significantly lower burnout levels than most groups that provided suggestions. The specific details are presented in Table 3.

**Table 3:** Univariate analysis of general characteristics of study participants and mean total burnout scores.

Variable	Category	Quantity (*N* = 1,520)	Number of people experiencing burnout	Percentage (%)	Overall Burnout Score ($\bar{X}$ ±*S* points)	Statistical measure (*t*/*F*)	*P*	*Post-hoc* test
Gender	Male①	522	338	64.75	1.78 ± 0.98	*t* = −1.773	0.076	–
	Woman②	998	657	65.83	1.87 ± 0.99	–	–	–
Age (years)	≤ 25①	182	124	68.13	1.86 ± 0.91	*F* = 3.852	< 0.001	⑦ < ①, ⑦ < ②, ⑦ < ③, ⑦ < ④, ⑦ < ⑤, ⑦ < ⑥
	26–30②	249	184	73.90	2.07 ± 1.05	–	–	② > ①
	31–35③	228	149	65.35	1.76 ± 0.96	–	–	–
	36–40④	300	197	65.67	1.90 ± 0.95	–	–	④ > ①
	41–45⑤	247	150	60.73	1.77 ± 1.01	–	–	–
	46–50⑥	137	90	65.69	1.71 ± 0.96	–	–	–
	>50 years⑦	177	101	57.06	1.70 ± 1.02	–	–	–
Full-time and Part-time Status	Full-time①	259	160	61.78	1.72 ± 0.93	*t* = −2.099	0.036	② > ①
	Part-time②	1,261	835	66.22	1.86 ± 1.00	–	–	–
Education	Bachelor's degree or higher①	501	333	66.47	1.92 ± 1.07	*t* = 2.210	0.027	–
	Associate degree or lower③	1,019	662	64.97	1.80 ± 0.95	–	–	–
Professional specialty	doctor①	654	409	62.54	1.75 ± 0.98	*F* = 2.517	0.040	–
	Nurse②	500	335	67.00	1.90 ± 0.98	–	–	② > ①
	Technician③	91	65	71.43	1.94 ± 0.91	–	–	–
	Pharmacist④	136	84	61.76	1.86 ± 1.06	–	–	–
	Other majors⑤	139	102	73.38	1.92 ± 1.04	–	–	⑤ > ①
Professional Title	Beginner①	1,177	775	65.85	1.82 ± 0.98	*F* = 0.809	0.445	–
	Intermediate②	245	150	61.22	1.90 ± 1.07	–	–	–
	Advanced③	98	70	71.43	1.90 ± 0.91	–	–	–
Years of work experience after graduation	≤ 5 years①	317	220	69.40	1.91 ± 0.96	*F* = 2.671	0.021	–
	6–10 years②	331	234	70.69	1.94 ± 1.02	–	–	–
	11–15 years③	296	183	61.82	1.80 ± 0.99	–	–	–
	16–20 years④	205	134	65.37	1.88 ± 0.96	–	–	–
	21–30 years⑤	241	150	62.24	1.70 ± 0.99	–	–	-
	>30 years⑥	130	74	56.92	1.69 ± 1.00	–	–	–
Years of experience in mental health prevention and treatment	≤ 5 years①	997	662	66.40	1.88 ± 0.99	*F* = 4.080	0.007	③ < ①
	6–10 years②	367	242	65.94	1.83 ± 0.97	–	–	–
	11–15 years③	111	65	58.56	1.61 ± 1.00	–	–	–
	≥16 years④	45	26	57.78	1.53 ± 0.86	–	–	–
Level of specialized prevention agency	Municipal①	48	21	43.75	1.58 ± 1.09	*F* = 3.677	0.026	–
	County-level②	146	103	70.55	2.00 ± 1.11	–	–	–
	Township-level③	1,326	871	65.69	1.83 ± 0.97	–	–	–
Work recommendations	No suggestions①	885	539	60.9	1.67 ± 0.88	*F* = 16.170	< 0.001	③, ④, ⑧ > ①;④ > ②, ⑤, ⑥, ⑦; ③ > ⑥, ⑦; ⑧ > ②, ⑥, ⑦
	Strengthen professional training②	138	88	63.77	1.88 ± 1.05	–	–	–
	Hire additional staff③	82	66	80.49	2.27 ± 1.07	–	–	–
	Reduce the workload④	137	114	83.21	2.43 ± 1.04	–	–	–
	Improve benefits⑤	65	41	63.08	2.01 ± 1.20	–	–	–
	Strengthen interdepartmental coordination⑥	86	55	63.95	1.83 ± 1.09	–	–	–
	Increase funding⑦	70	47	67.14	1.83 ± 0.91	–	–	–
	Reduce the difficulty of the task⑧	57	45	78.95	2.28 ± 0.96	–	–	–

### 3.4 Multivariate logistic regression analysis of job burnout

The results of the multivariate binary logistic regression in this study indicate that, compared to workers at municipal-level mental health institutions, those at county- and township-level institutions face a significantly higher risk of occupational burnout; The risk of occupational burnout was significantly higher among mental health workers who expressed a desire to “increase staffing,” “reduce workload,” or “lower job difficulty.” The effects of other variables, such as demographic characteristics, years of service, and professional specialty, on occupational burnout were not statistically significant (*P* > 0.05). In the binary logistic regression analysis of this study, the overall model was significant (χ^2^ = 83.625, *P* < 0.001), and the Hosmer-Lemeshow test indicated a good model fit (χ^2^ = 7.960, *P* = 0.437). Using workers at municipal-level specialized prevention agencies as the reference group, workers at county-level specialized prevention agencies (OR = 2.977, 95% CI: 1.480–5.988, *P* = 0.002) and township-level mental health prevention agency staff (OR = 2.312, 95% CI: 1.210–4.416, *P* = 0.011) both had significantly increased risks of occupational burnout; work-related suggestions were also independent risk factors. Compared with mental health workers who made “no suggestions,” those who proposed “increasing staff” (OR = 2.714, 95% CI: 1.527–4.822, *P* < 0.001), “reduce workload” (OR = 3.449, 95% CI: 2.142–5.554, *P* < 0.001), and “reducing job difficulty” (OR = 2.745, 95% CI: 1.406–5.359, *P* = 0.003) showed a significantly higher risk of occupational burnout. See Table 4 for details and see Table 5 for variable coding.

**Table 4:** Multivariate binary logistic regression results of job burnout among mental health prevention workers.

Variable		*B*	S.E.	Wald	*P*	OR (95% CI)
Gender	Male (for reference)	–	–	–	–	–
	Woman	0.003	0.133	0.001	0.981	1.003 (0.774–1.301)
Age (years)	≤ 25 (for reference)	–	–	–	–	–
	26–30	0.204	0.251	0.661	0.416	1.227 (0.749–2.008)
	31–35	−0.158	0.285	0.309	0.578	0.853 (0.488–1.492)
	36–40	−0.138	0.302	0.209	0.648	0.871 (0.482–1.574)
	41–45	−0.348	0.332	1.101	0.294	0.706 (0.369–1.352)
	46–50	−0.099	0.377	0.069	0.793	0.906 (0.433–1.896)
	>50	−0.315	0.420	0.563	0.453	0.730 (0.320–1.662)
Full-time and part-time status	Full-time (for reference)	–	–	–	–	–
	Part-time	0.182	0.161	1.264	0.261	1.199 (0.874–1.645)
Education	Bachelor's degree or higher (for reference)	–	–	–	–	–
	Associate degree or lower	−0.081	0.133	0.378	0.539	0.922 (0.711–1.195)
Professional specialty	Doctor (for reference)	–	–	–	–	–
	Nurse	0.126	0.150	0.708	0.400	1.135 (0.845–1.523)
	Technician	0.250	0.261	0.921	0.337	1.284 (0.770–2.141)
	Pharmacist	0.019	0.209	0.009	0.926	1.020 (0.677–1.536)
	Other majors	0.209	0.214	0.952	0.329	1.232 (0.810–1.873)
Years of work experience after graduation	≤ 5 years (for reference)	–	–	–	–	–
	6–10 years	−0.001	0.226	0	0.995	0.999 (0.642–1.554)
	11–15 years	−0.310	0.267	1.349	0.245	0.734 (0.435–1.237)
	16–20 years	−0.091	0.303	0.091	0.763	0.913 (0.504–1.653)
	21–30 years	−0.195	0.328	0.353	0.553	0.823 (0.433–1.565)
	>30 years	−0.357	0.421	0.721	0.396	0.700 (0.307–1.596)
Years of experience in mental health prevention and treatment	≤ 5 years (for reference)	–	–	–	–	–
	6–10 years	0.078	0.144	0.291	0.589	1.081 (0.815–1.432)
	11–15 years	−0.079	0.225	0.124	0.725	0.924 (0.595–1.436)
	≥16 years	0.105	0.346	0.091	0.762	1.110 (0.564–2.187)
Level of specialized prevention agency	Municipal epidemic prevention (for reference)	–	–	–	–	–
	County-level mental health prevention	1.091	0.357	9.355	0.002	2.977 (1.480–5.988)
	Township-level precision prevention	0.838	0.330	6.437	0.011	2.312 (1.210–4.416)
Work recommendations	No recommendations (for reference)	–	–	–	–	–
	Strengthen professional training	0.209	0.197	1.131	0.287	1.233 (0.838–1.813)
	Hire additional staff	0.998	0.293	11.578	< 0.001	2.714 (1.527–4.822)
	Reduce the workload	1.238	0.243	25.939	< 0.001	3.449 (2.142–5.554)
	Improve benefits	0.257	0.28	0.842	0.359	1.293 (0.747–2.237)
	Strengthen interdepartmental coordination	0.314	0.248	1.606	0.205	1.369 (0.842–2.226)
	Increase funding	0.340	0.271	1.576	0.209	1.404 (0.827–2.386)
	Reduce the difficulty of the task	1.010	0.341	8.754	0.003	2.745 (1.406–5.359)

**Table 5:** Assignment of independent variables in binary logistic regression analysis.

Variable	Coding
Gender	Male = 1, woman = 2
Age (years)	≤ 25 = 1, 26–30 = 2, 31–35 = 3, 36–40 = 4, 41–45 = 5, 46–50 = 6, >50 = 7
Full-time and part-time status	Full-time = 1, part-time = 2
Education	Bachelor's degree or higher = 1, associate degree or lower = 2
Field of study	Doctor = 1, nurse = 2, technician = 3, pharmacist = 4, other majors = 5
Years of work experience after graduation	≤ 5 years = 1, 6–10 years = 2, 11–15 years = 3, 16–20 years = 4, 21–30 years = 5, >30 years = 6
Years of experience in mental health prevention and treatment	≤ 5years = 1, 6–10 years = 2, 11–15 years = 3, ≥16 years = 4
Level of specialized prevention agency	Municipal = 1, county-level = 2, township-level = 3
Work recommendations	No suggestions = 0, strengthen professional training = 1, hire additional staff = 2, reduce workload = 3, improve compensation = 4, strengthen interdepartmental coordination = 5, increase funding = 6, reduce the difficulty of the work = 7

## 4 Discussion

In recent years, as healthcare reforms in China have continued to advance, medical institutions at the municipal, county, and township levels have been presented with new opportunities for development, while being entrusted with additional responsibilities and tasks. In particular, county-level and township health institutions are responsible for mental health prevention and treatment as well as for the basic diagnosis and treatment of common and prevalent diseases, chronic disease management, and family doctor contract services. At the same time, they must cope with various burdensome inspections, supervision, and reporting requirements, resulting in immense work pressure and a high risk of severe occupational burnout (26). Data from this study indicate that in December 2024, the overall prevalence of occupational burnout among mental health workers in Guangxi reached 65.46%, with 56.05% of individuals exhibiting mild occupational burnout and 9.41% experiencing moderate to severe occupational burnout. The average burnout score for all study participants was 1.83 ± 0.99, indicating mild occupational burnout. Other Chinese scholars have also found high rates of occupational burnout among mental health professionals in their studies, such as 70.8% in Beijing (27), 79.2% in Qinghai Province (28), and 69.6% in Xiamen (29). In addition to potentially leading to decreased patient satisfaction, increased medical errors, and reduced quality of doctor-patient communication (30, 31), it may also lead to a decline in the physical functioning of mental health professionals (32) and a loss of talent, ultimately resulting in instability within the mental health workforce—a situation that warrants our further attention and concern.

In this study, we found that the level of mental health institutions and mental health workers' suggestions regarding “increasing staff,” “reducing workload,” and “lowering the difficulty of tasks” were key factors influencing occupational burnout. Compared with municipal-level mental health workers, both county-level and township-level mental health workers exhibited significantly higher risks of occupational burnout, with the risk among county-level staff being particularly pronounced (OR = 2.977, 95% CI: 1.480–5.988, *P* = 0.002). The burnout rate reached 70.55%, higher than the 65.69% observed at the township level and 43.75% at the municipal level. Previously, it was widely believed that burnout levels were generally higher among primary-level health service workers, and research by many scholars has focused on this group (33–35), often neglecting the population in county-level institutions.

However, this study may offer a new perspective: county-level agencies may be at the intersection of multiple pressures, as they must meet public health service targets set by municipal authorities and guide the frontline work of township-level agencies. The combination of these dual management pressures, coupled with relatively insufficient resource support, places them at a higher risk of burnout. This view is consistent with the findings of other Chinese scholars who have studied the working mechanisms of county-level governments. In recent years, as national resources have been decentralized to lower levels, an increasing number of departmental tasks have been transformed into core responsibilities at the county level. County-level governments must not only cope with complex performance evaluations from higher authorities but also coordinate and oversee frontline implementation at the township level. This multi-headed management and the decentralization of responsibilities have led to a governance state characterized by heavy workloads and low efficiency (36), which easily results in an overload of tasks for county-level government departments (37).

In addition, work-related suggestions are significant predictors of occupational burnout. Compared to mental health workers who reported “no suggestions,” those who expressed a desire to “reduce workload,” “increase staffing,” or “lower the difficulty of work” showed a significantly higher risk of burnout. Among these, the impact of “reducing workload” was the most pronounced (OR = 3.449, 95% CI: 2.142–5.554, *P* < 0.001), with a burnout rate reaching 83.21%. In recent years, owing to the continuous advancement of China's Project 686 and the Chinese government's emphasis on patients with severe mental disorders, efforts to diagnose and treat suspected cases have been carried out in communities across the country. The number of patients registered in China's National Severe Mental Disorders Information System increased from 4,297,363 in 2014 to 6,430,587 in 2020 (38, 39), representing a 33.17% increase. The rate of standardized patient management increased from 35.77% to 89.01%, and the rate of regular medication adherence increased from 24.31% to 68.84%. Significant progress has been made in patient service management, resulting in more stable patient conditions and a year-on-year decrease in cases of violence and fatalities involving patients. However, this means that without an increase in staff, they must serve more patients than before while maintaining a higher standard of care—a situation that aligns with the recommendations of mental health professionals to increase staffing and reduce workloads. Furthermore, to prevent patients from harming others, comprehensive community service management must be implemented in collaboration with judicial, public security, and neighborhood (town) authorities for high-risk and very high-risk patients (40). However, due to the sense of self-shame associated with mental illness (41), many patients and their families refuse to let others know of their condition and are even more resistant to home visits by mental health workers. They often shut the door in workers' faces, deliberately avoid them, and even resort to verbal abuse and threats. This inadvertently increases the difficulty of the work and exacerbates the occupational burnout experienced by mental health workers.

As shown in Table 2 and Figure 1, the levels of occupational burnout among mental health workers in the four cities of Laibin, Yulin, Hezhou, and Nanning in Guangxi Province were higher than those in other cities. According to the December 2024 report from the National Severe Mental Disorders Information System, Yulin City ranked first in Guangxi in terms of the reported prevalence of severe mental disorders. This implies that mental health administrators in this city must serve and follow up with more patients than those in other regions, which may have contributed to the higher levels of occupational burnout among mental health workers in this area. In contrast, Laibin City and Hezhou City ranked first and second, respectively, among all cities in Guangxi for standardized patient management rates, at 98.45% and 98.12%. Additionally, Laibin City ranked first, second, and second, respectively, among all cities in Guangxi for patient medication adherence, physical examination rates, and face-to-face visit rates. It is worth noting that Hezhou City ranks fourth from the bottom in overall development in Guangxi, while Laibin City ranks second from the bottom. However, patient management in these two cities ranks among the top in Guangxi. This implies that they face heavier workloads and must exert greater effort, which may be the reason for their higher levels of occupational burnout. As the political, economic, and cultural center of Guangxi, Nanning has a large patient population and high population mobility. To prevent patients from developing symptoms that could disrupt social stability, mental health workers must devote significant effort to patient follow-up and service management. We believe that this is the reason for the higher levels of burnout among mental health workers in this region. Therefore, government officials should pay close attention to the physical and mental wellbeing of mental health workers in these four cities, and the stability of their workforce.

Burnout not only reduces the work efficiency and quality of mental health professionals but also negatively impacts their quality of life and hinders their career development. We urge the relevant authorities to remain highly vigilant and give this issue the attention it deserves, promptly identifying problems and addressing urgent concerns through targeted measures to ensure the healthy and sustainable development of mental health professionals.

This study also has certain limitations. First, as a cross-sectional study, it is difficult to establish clear causal relationships between variables, and the findings are susceptible to recall bias. Regarding sample representativeness, although approximately 96.55% of mental health professionals in Guangxi completed the questionnaire, the study did not achieve full coverage. Additionally, during the survey process, respondents may have intentionally or unintentionally exaggerated their views in order to improve relevant institutions or personnel, thereby leading to reporting bias. Finally, this study reflects only the current situation in Guangxi. Economic development levels, staffing arrangements, and work requirements vary across regions. Therefore, these conclusions may not be generalizable to other regions of China or globally. When applying these findings to other regions, local socioeconomic and cultural factors should be carefully considered to ensure appropriate adjustments and interpretations.

## 5 Conclusion

This study found that, in December 2024, occupational burnout among mental health professionals in Guangxi was at a relatively high level. Mental health professionals at the county and township levels were at a higher risk of occupational burnout; whereas, those working in municipal-level institutions exhibited a lower risk of occupational burnout. Analysis of the suggestions provided by mental health workers at all levels indicates that those who called for “increasing staff,” “reducing workload,” and “lowering work difficulty” had a higher risk of occupational burnout. Among all cities in Guangxi, mental health workers in Laibin, Yulin, Hezhou, and Nanning exhibited higher levels of occupational burnout than those in other cities. To ensure the stability of the mental health workforce and improve service management for patients with severe mental disorders, we believe that the supervisory agencies and relevant departments overseeing mental health workers must take responsive measures. These include increasing staff allocations, appropriately lowering performance metrics, and enhancing interdepartmental coordination to reduce the complexity of work. Such efforts are essential for achieving efficient, orderly, and sustainable operations of the mental health workforce, which plays an irreplaceable role in safeguarding their wellbeing.

## Data Availability

The datasets generated and analyzed during the current study are available from the corresponding author upon reasonable request and with appropriate approval.
